# Evaluation of the Validity of Digital Optical Microscopy in the Assessment of Marginal Adaptation of Dental Adhesive Interfaces

**DOI:** 10.3390/polym14010083

**Published:** 2021-12-27

**Authors:** René Daher, Ivo Krejci, Enrico di Bella, Laurine Marger

**Affiliations:** 1 Division of Cariology and Endodontology, University of Geneva, University Clinics of Dental Medicine, 1, Rue Michel Servet, 1204 Geneva, Switzerland; ivo.krejci@unige.ch; 2 Department of Political Science, University of Genoa, 16124 Genoa, Italy; enrico.dibella@unige.it

**Keywords:** marginal adaptation, dental adhesive, optical microscope, scanning electron microscope

## Abstract

Analysis of marginal adaptation of dental adhesive interfaces using scanning electron microscopy has proven to be a powerful nondestructive method to evaluate the quality of adhesion. This methodology is, however, time-consuming and needs expensive equipment. The purpose of this study was to evaluate the possibility and efficiency of using a digital optical microscope (DOM) to perform marginal analysis and to compare it with the scanning electron microscope (SEM) analysis. Fifteen defect-free molars were selected for this study. Class V cavities were prepared and restored with resin composite, and epoxy replicas were obtained from silicone impressions of the restored teeth. Custom-made image analysis software was then used to measure the percentage of the noncontinuous margins (NCM) of each sample. To compare the DOM to the gold standard, SEM, each sample was analyzed 10 times using the DOM and three times using the SEM, by the same experienced operator. The repeatability coefficient and concordance were evaluated, and a Bland and Altman analysis was used for comparison of the two methods of measurements. To validate the DOM analysis method, an ANOVA approach (Gage R R) was used. Repeatability and reproducibility, which are two components of precision to validate the DOM analysis system, were calculated. For this, the same restorations were analyzed by two additional operators three times with the DOM. The duration of each step of the analysis using both methods was also recorded as a secondary outcome. Regarding the repeatability of each method, the Friedman test showed no statistically significant difference within the repetitions of measurements by SEM and DOM (*p* = 0.523 and *p* = 0.123, respectively). Moreover, the Bland-Altman analysis revealed a bias of 0.86 and concluded no statistically significant difference between the two methods, DOM and SEM. ANOVA evaluated DOM measurement system variation including the variances of repeatability and reproducibility that represented, respectively, 0.3% and 4% of the variance components. Reproducibility or inter-operator variability represented the principal source of variability with a statistically significant difference (*p* = 0.024). The time required for analysis with SEM was almost double that of DOM. The use of digital optical microscopy appears to be a valid alternative to the SEM for the analysis of marginal adaptation of dental adhesive interfaces. Further studies to evaluate the effect of training of operators in digital optical microscopy could reveal higher accuracy for this method and inter-operator agreement when experience is gained.

## 1. Introduction

Dental resin adhesives continue to evolve progressively, and new products are released almost every year. Despite all the clinical advantages that adhesive restorations present, such as reduction of unnecessary tooth mutilation and simpler clinical procedures [1], an insufficient seal due to multiple factors remains one of the current drawbacks. The main limitation of current dental adhesives is not the lack of retentive capacity, but rather the quality of the bond throughout the interface. Polymerization shrinkage is usually the first challenge to the adhesive layer, which induces stresses that can locally generate micro-gaps [2]. Whether direct or indirect restorations, aging factors such as water, mechanical loads and staining elements have an important effect on the adhesive interface, which often leads to a worsening of the integrity of that margin from an esthetic and a biomechanical point of view [3,4]. Such changes do not necessarily translate into debonding of the restoration, especially in newer generations of adhesive systems, but they constitute a major reason for retreatment due to secondary caries or appearance-related clinical failures [5,6,7]. Therefore, among the multiple tests that exist to evaluate the performance of adhesive systems, marginal integrity assessment appears to be relevant for the future, rather than bond strength tests that continue to report difficult-to-relate-to values, between adhesives that rarely fully debond clinically [8,9,10,11].

The quality of an adhesive interface can be assessed either qualitatively, describing its leakage and infiltration extent using dye penetration tests, or by quantitative techniques to report ill-adapted margins. Regarding the quantitative evaluation of marginal adaptation, multiple methods have been used, such as flow measurements [12] or imaging tools such as micro-computed tomography (micro-CT) [13] and scanning electron microscopy (SEM) [12,13,14]. SEM marginal analysis is currently the most commonly used method since it is nondestructive and thus allows, at multiple time intervals, to accurately measure and calculate the percentages of closed margins which represent perfect adaptation of the adhesive restoration. This consists of creating replicas of the samples, usually before and after thermo-mechanical aging, then analyzing the entire margin under high magnifications [15,16].

Digital optical microscopes (DOM) are becoming more accessible and present a powerful and more economical alternative to SEM in less demanding tasks. According, to the authors’ knowledge, the use of DOMs as a mean to perform marginal analysis of adhesive interfaces has not been described in the literature so far.

In this context, the objectives of the present study were to compare the efficiency of this DOM method to the gold standard method (SEM) and to assess the validity of using DOM to perform marginal analysis. To validate this suggested workflow, the repeatability of marginal analysis through DOM, which represents the first source of variation due to the DOM measurement device, had to be first evaluated and then compared to the results obtained by the gold standard SEM method. The second step was to evaluate the second source of measurement system variation, reproducibility, which is the capacity to scale the new DOM method to new operators (interoperator variability). Repeatability and the reproducibility are two components of precision that are required to validate the DOM method.

## 2. Materials and Methods

Fifteen freshly-extracted intact human third molars were used in this study. The teeth were stored in 0.1% thymol solution until the time of the experiment. Buccal class V cavities were prepared, with their coronal margins in enamel and the cervical margins in dentin, and adhesive composite restorations were readied. Restorations were polished with flexible discs (SofLex, 3M, St. Paul, MN, USA). As the goal of this study was not to compare adhesive techniques nor materials, random combinations of these two were used to restore the cavities and were obscured from the investigators in order to avoid bias during the analysis. Two brands of universal adhesives were used in a self-etch mode, and one brand of three-step etch and rinse system was also used. The cavities were then restored with either a nano-hybrid or a micro-hybrid composite material. Using multiple adhesive techniques and materials was done to cover the range of possible observations that would occur in such marginal analysis studies. After completion of the polishing procedure, the teeth were cleaned with prophylactic nylon brushes and toothpaste, and impressions with a polyvinylsiloxane material (President light body, Coltène-Whaledent, Altstätten, Switzerland) were taken of each restoration. A slow-curing transparent epoxy resin system for embedding and impregnation of materialographic specimens (EpoFix Resin, Struers, Rødrove, Denmark) was used to fabricate the replicas. Fifteen parts of EpoFix Resin (bisphenol-A-(epichlorhydrin) average molecular weight ≤ 700, oxirane, mono[(C12-14-alkyloxy)methyl] derivs) were mixed with two parts of EpoFix hardener according to the manufacturer’s proportions, and the mixture was then poured into the silicone impressions to obtain one replica for each of the fifteen teeth, and these were subsequently gold-coated after the complete hardening of the resin.

### 2.1. Marginal Analysis

**Figure 1:**
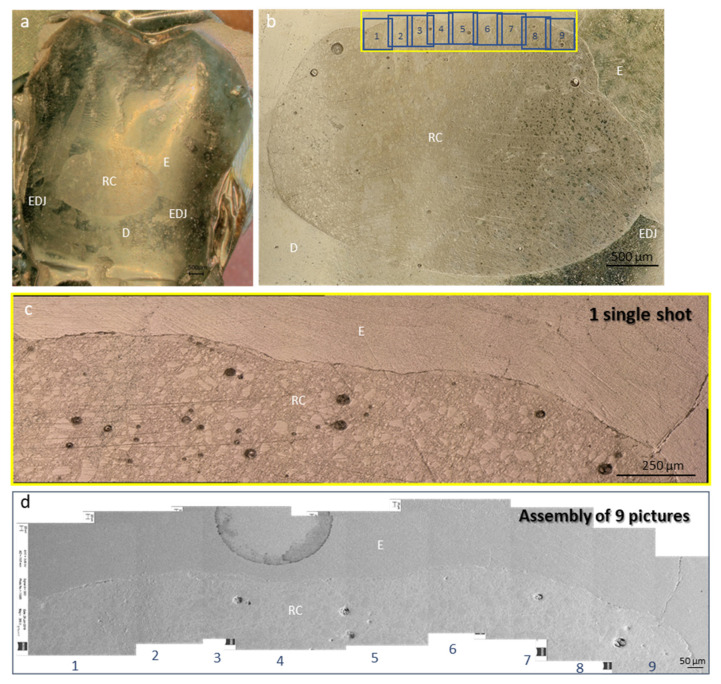
(**a**) Photograph of one the fifteen gold-coated tooth replicas with a class V composite restoration, (**b**) DOM image of the entire cavity at 50× magnification and (**c**) a semiautomatically generated assembly at 200× showing the enamel part in the yellow box. Only one capture was required to visualize this entire portion of the cavity, which represents a quarter of the analyzed interface. (**d**) Nine successive SEM images at 200× magnification, with the outlines presented in 1b and numbered from 1 to 9, were manually assembled to visualize the same region of interest. RC resin composite, E enamel, D dentin, EDJ enamel dentin junction.

For each sample, images of the entire marginal contour at a 200× magnification were taken using both a scanning electron microscope (SEM) (Sigma 300VP, Zeiss, Oberkochen, Germany) and a digital optical microscope (DOM) (Keyence VHX-5000, Keyence International, Mechelen, Belgium). For the SEM, it was required to manually navigate through the whole margin at 200× and to manually assemble the single images (Figure 1), while for the DOM, a single maneuver by delimiting the region of interest and the required magnification of 200× was required to generate the whole assembly (Figure 1). The time required to analyze each sample was recorded to compare the efficiency of both methods. The same sets of images were used by all of the investigators. Custom-made image analysis software (Marginal Analysis 4.0, RD, Geneva, Switzerland) was then used to measure the percentage of noncontinuous margins (%NCM) of each sample. This percentage represents the length of the nonadapted segments of the tooth/restoration interface over the total length of the margin. A portion was considered noncontinuous if the transition between the two substrates was not perfectly adapted.

### 2.2. Comparison of Optical Microscopy and Scanning Electron Microscopy Methods (OM/SEM)

To evaluate the precision or repeatability of both DOM and SEM analysis methods, a single experienced operator (OP-A), analyzed all samples ten times using the DOM and three times using the SEM. Given that SEM is the reference method, three repetitions were sufficient. A break of 15 min was taken after each three samples, and a minimum of 24 h was allowed to elapse between the repetitions of the samples. The process was completely blinded. Repeatability of the measures and dispersion of the results were then assessed by calculating the coefficient of variability (CV), which is the ratio of the standard deviation (σ) over the mean (µ), CV = σ/µ.

To evaluate the trueness or the validity of the values obtained through the optical microscopy method, the SEM method was used as a baseline and the results of the DOM analysis were compared with those of the SEM analysis by the same operator OP-A. A Bland and Altman graph was plotted to evaluate the concordance and the agreement limits of the two methods [17].

### 2.3. Interoperator Variability

**Figure 2:**
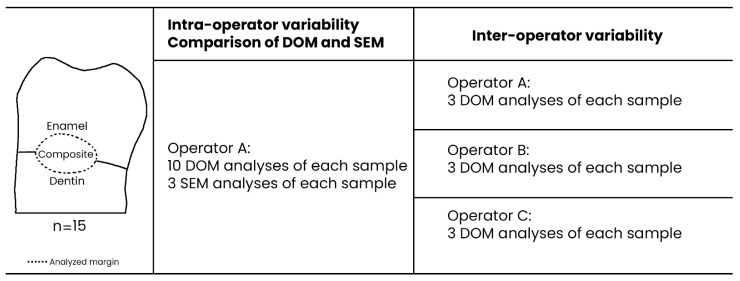
Representation of the study design.

Two additional operators (OP-B and OP-C) analyzed three times the same sets of images using OM on the same 15 samples to measure e reproducibility. OP-B and OP-C were experienced with the SEM marginal analysis method but only had one day of prior training on the OM marginal analysis method. These selection criteria of the additional operators were chosen to avoid any prior experience effect on the results of the analysis with DOM. Each operator performed the repetitions alone and in a blinded fashion, respecting the previously mentioned analysis conditions. A schematic representation of the study design is presented in Figure 2.

### 2.4. Statistical Analyses

A two-way analysis of variance (ANOVA) was used to identify the two sources of variability (repeatability, i.e., the method through optical microscopy, and reproducibility, i.e., the operators) and their interactions. This allowed quantification of the variance of the repeatability inherent to the measuring device, and the reproducibility evaluated by having multiple operators using the same measurement methods. Repeatability and reproducibility are two components of precision to validate a measurement system. The normality of the within-cell residuals was checked using the Shapiro-Wilk normality test.

To validate the OM analysis method, an ANOVA approach (Gage R&R) was used. First the percentage of the total variance (% GRR) due to the measurement system was evaluated. The total variance was used to determine the contribution of each source of the OM measurement system including its repeatability and reproducibility. The percentage of GRR was determined by dividing the variance for each source of the total variance. To evaluate and validate our measurement system, and according to the AIAG guidelines, the acceptance criteria for the measurement system are (i) if the percentage of the total variance (% GRR) is less than 1%, the measurement system is acceptable; (ii) if the % GRR is between 1 to 9%, the measurement system is acceptable, but some modifications are necessary to improve the system, and (iii) if the percentage is over 9%, the system is considered unacceptable.

The repeatability coefficient and concordance were evaluated, and a Bland and Altman analysis was used for comparison of the two methods of measurements. The repeatability and the reproducibility, which are two components of precision to validate the OM analysis system, were calculated. For this, the same restorations were analyzed by two additional operators three times with the OM.

Repeatability, due to the violation of some normality assumptions, was evaluated using the Friedman test.

All statistical analyses were run with the XLSTAT (Addinsoft, New York, NY, USA) statistical add-in of Excel. The repeatability measures were checked for normality using the Shapiro-Wilk and Grubb test, and the significance level of statistical tests was set to *p* = 0.05.

## 3. Results

The mean durations for each step of the analysis procedure are presented in Table 1.

**Figure 3:**
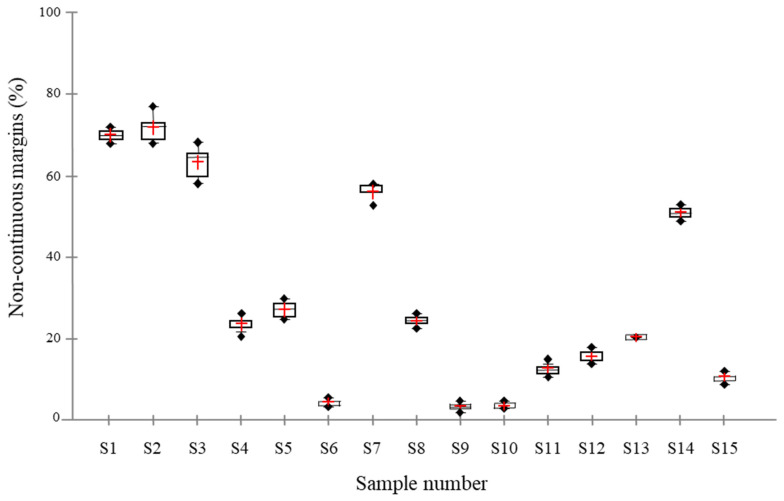
Box plot showing the percentage of noncontinuous margins (% NCM) for each of the fifteen class V cavities (S1–S15, S: samples) from pictures recorded with optical microscopy. The marginal analysis was repeated ten times with the same operator. Red cross: mean, dashed line: median, ⬩: Outliers.

The results of the marginal analysis of OP-A using DOM expressed as the mean percentages of noncontinuous margins (% NCM) are detailed in Figure 3. These values were clearly heterogeneous between the fifteen samples and ranged from 4% (i.e., sample 9) to 72% (i.e., sample 2). This variation was introduced deliberately by diversifying restorative techniques and materials to widen the scope of the analysis methods. Normality tests revealed a normal distribution among the ten repetitions of all samples, except for samples 6, 10, 12 and 13.

### 3.1. Comparison of the DOM Method with the Gold Standard SEM

For repeatability, Friedman test results showed no statistically significant difference among the ten repetitions of the DOM group (*p* = 0.123) nor among the three repetitions of the SEM group (*p* = 0.523). The coefficient of variation was smaller for the DOM group (0.075 ± 0.05) than for the SEM group (0.11 ± 0.07).

**Figure 4:**
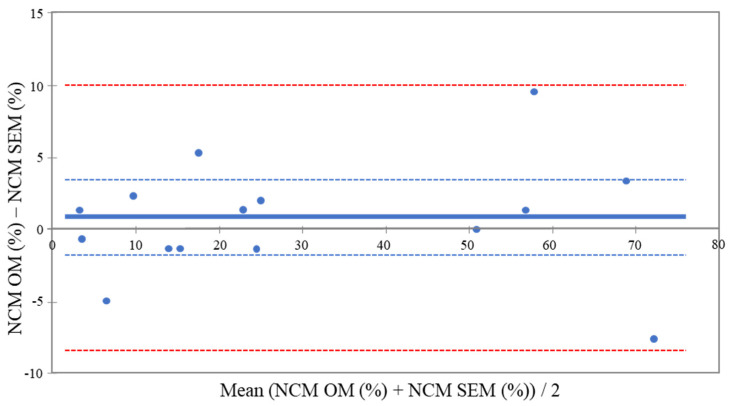
Bland and Altman plot showing the concordance between marginal analysis of the two methods. The mean difference between the methods is plotted with the solid blue line against the averages of the two methods and represents the bias. The blue dotted lines are +/− 95% confidence interval and the red dotted lines represent the limits of agreement. The majority of the observations are within the limits of agreement.

The concordance results between DOM and SEM are presented in the Bland and Altman graph (Figure 4). The bias of 0.86 that was observed between both methods, and that lies within the 95% confidence interval (−1.74; 3.47), indicates no statistically significant difference between DOM and SEM (Figure 3).

### 3.2. Analysis of the DOM Method Using the Two-Way Crossed ANOVA Analysis

**Figure 5:**
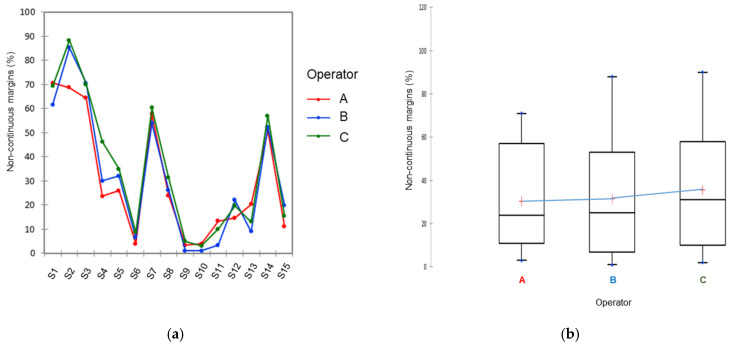
(**a**) Representation of the differences in the marginal analysis (percentage of noncontinuous margins) between the three operators (A, B and C) for all the studied samples (S1–S15, S: sample). (**b**) Box plot showing the variance between operators. The difference between operators is represented by the linearity of the blue line. Operator C has a tendency to overestimate %NCM compared to the two other operators. Horizontal line: median. Boxes: 25–75%. Bars: range of non-outliers.

Results of reproducibility analysis are shown in Figure 5a, which represents the variability of the measures made by the three operators on the 15 samples. The analysis by the three operators was identical for some samples, such as samples 6 and 7, and different for other samples, such as sample 4. The analysis allows evaluation of the magnitude of errors in the measurements. Interoperator variability is presented in Figure 5b. The variance between operators is considered low, but still statistically different (*p* = 0.024) (Table 2), with higher values for operator C compared to the two other operators (Figure 5b).

Operator-related variability was low and contributed to 4.01% of total variance compared to the more significant contribution of the sample variability to the overall variability of the system (95.66%) (Table 2). The three operators obtained repeatable results with 0.33% of total variance. The measurement DOM method variation that is the sum of the repeatability and reproducibility variance components represented 4.34% of the process variation (Table 2).

## 4. Discussion

The objective of the ANOVA method is to determine the viability of the tested DOM measurement system by quantifying the interaction between repeatability and reproducibility. Acceptance criteria for the ANOVA method are divided into three class: under 1%, it is an adequate measurement system; 1% to 9%, it is acceptable with modifications; and over 9% it is considered to be unacceptable [18]. The current results of 4.34% fall in the class of acceptable with some modifications required. In the present study, it was observed that interoperator agreement was the main element that affected this drawback, and training of more than one day should be recommended to become familiar with the visualization of different margin characteristics using DOM, which could appear slightly different than on SEM.

Alongside mechanical properties and esthetics, marginal adaptation is a crucial element in the overall success of dental restorations. This important factor has a direct effect on the biological behavior and aspects of the tissues surrounding any restoration margin. A flaw at this level could retain plaque, colorants, and impede oral hygiene maintenance in this concerned region. All of these could eventually lead to clinically relevant complications such as caries, discolored margins and inflamed soft tissues [19].

Marginal integrity is generally assessed either visually using magnification aids or using a dental probe at the clinical level. To evaluate the quality of the seal at the marginal level, dye penetration is one of the most used destructive methods [20] since it requires sectioning of the samples, which could limit comparisons at different time points of the experiment. Microcomputed tomography (micro-CT) is usually considered a nondestructive method that gives a very detailed view of both external and internal topology of an adhesive interface [21]. Nevertheless, it requires prolonged and costly scanning time per sample, which is often required to be made in a dry situation. In the case of adhesion testing, keeping the teeth dry for around one hour could compromise the adhesion and could therefore classify the test as destructive [22]. The replica technique [23], described and used in the present study, is an accessible nondestructive method that has proven to be reliable, especially for marginal analysis, to directly compare adhesive systems in a controlled setup [23]. Limitations of using a scanning electron microscope are the price and availability of this microscope in laboratories, and the workflow is often slowed down by the required vacuum procedures and navigation through the samples, as seen in Table 1. Digital optical microscopes are, in general, cheaper than SEM, and present a less-demanding workflow.

Optical microscopes have existed for centuries, but the quality of imaging and especially the depth of field, has been enormously boosted by the introduction of digital optical microscopes. To validate the use of digital optical microscopy in the specific case of assessing the quality of marginal adaptation of adhesive dental restorations, three main tests were used in the present study. The first repeatability or precision test showed that the variability between the repeated analyses was not significant. Multiple types of adhesive restorations were included in this study to obtain a diversified population that would cover a wide range of possible values of %NCM [24,25]. For the second test comparing DOM to SEM, the latter was used as a gold standard because it has been the method of choice in hundreds of publications on the subject and is recognized as a valid method for marginal analysis. The results of this test showed that the concordance between DOM and SEM is very high and that both methods have statistically the same trueness with a slight overestimation of % NCM with DOM (positive bias of 0.86). The final test was to make sure that the DOM method can be taught to and reproduced by multiple operators. The results showed that there was a high agreement between operators who had never used this method before, and the main operator who had used it for a longer time. It was also noticed during the study that the time required to analyze one sample using the DOM was considerably shorter than the time needed for the analysis of the same sample in the SEM (Table 1).

One possible limitation of the present study is the use of a few combinations of adhesives and restorative materials. Nevertheless, the use of different brands and types of such materials is not expected to influence the visualization of the margins under the microscope. In future studies, it would be interesting to evaluate the performance of other ranges of digital optical microscopes, such as more accessible models.

## 5. Conclusions

This study shows that digital optical microscopy can be considered a promising, reliable and repeatable method to perform marginal analysis of adhesive dental restorations using the replica technique. Having this time and cost-effective method can be beneficial in the assessment of newer generations of adhesive systems and techniques. The results also showed that the quality of analysis done by this method is comparable to the current gold standard method using scanning electron microscopy. Even though new users of the DOM analysis technique were able to obtain valid results after one day of training, additional experience may be needed to increase the concordance between operators.

## Figures and Tables

**Table 1:** Required time (±SD) for each step of the marginal analysis procedure calculated as a mean value from the fifteen evaluated samples.

Time/Sample (min)	DOM	SEM
Preparation (replica, coating)	30 ± 2	30 ± 2
Recording	19 ± 6	36 ± 11
Assembly	Semi-automatic —	Manual 12 ± 3
Marginal analysis	10 ± 3	22 ± 7
Total	59	100

**Table 2:** Two-way ANOVA table with interaction. Degree of freedom (DF), sum of squares (SS), variance (V), Fisher-test (F), *p*-value (P), percentage of overall variation from each component (% Contribution), *p* < 0.05.

Source	DF	SS	V	F	P	% Contribution
Repeatability	90	212.66	2.36	90		0.33
Reproducibility	2	624.99	28.77	4.29	0.024	4.01
Total GRR (DOM measurement system)			31.13			4.34
Part-to-Part (Samples)	14	87,512.54	686.46	85.98	<0.0001	95.66
Total Variation	134	90,385.65	717.60			100

## Data Availability

Not applicable.
